# Views of Caregivers on the Ethics of Assistive Technology Used for Home Surveillance of People Living with Dementia

**DOI:** 10.1007/s12152-017-9305-z

**Published:** 2017-01-24

**Authors:** Maurice Mulvenna, Anton Hutton, Vivien Coates, Suzanne Martin, Stephen Todd, Raymond Bond, Anne Moorhead

**Affiliations:** 1 School of Computing and Mathematics, Ulster University, Newtownabbey, UK; 2 School of Creative Arts and Technologies, Ulster University, Newtownabbey, UK; 3 School of Nursing, Ulster University, Newtownabbey, UK; 4 School of Health Science, Ulster University, Newtownabbey, UK; 5 Western Health and Social Care Trust, Altnagelvin Hospital, Londonderry, UK; 6 School of Communication, Ulster University, Newtownabbey, UK

**Keywords:** Independent living, Autonomy, Assistive technology, Ethics, Dementia, Alzheimer’s disease, Cameras, Video, Surveillance

## Abstract

This paper examines the ethics of using assistive technology such as video surveillance in the homes of people living with dementia. Ideation and concept elaboration around the introduction of a camera-based surveillance service in the homes of people with dementia, typically living alone, is explored. The paper reviews relevant literature on surveillance of people living with dementia, and summarises the findings from ideation and concept elaboration workshops, designed to capture the views of those involved in the care of people living with dementia at home. The research question relates to the ethical considerations of using assistive technologies that include video surveillance in the homes of people living with dementia, and the implications for a person living with dementia whenever video surveillance is used in their home and access to the camera is given to the person’s family. The review of related work indicated that such video surveillance may result in loss of autonomy or freedom for the person with dementia. The workshops reflected the findings from the related work, and revealed useful information to inform the service design, in particular in fine-tuning the service to find the best relationship between privacy and usefulness. Those who took part in the workshops supported the concept of the use of camera in the homes of people living with dementia, with some significant caveats around privacy. The research carried out in this work is small in scale but points towards an acceptance by many caregivers of people living with dementia of surveillance technologies. This paper indicates that those who care for people living with dementia at home are willing to make use of camera technology and therefore the value of this work is to help shed light on the direction for future research.

## Introduction

The number of older people (those aged 60 years or over) has increased substantially in recent years in most countries and this ageing population is projected to continue accelerating in coming decades [1]. By 2050, the global population of older persons is projected to more than double its size in comparison to 2015 demographics [1]. As a consequence, there will be a significant burden on healthcare services to treat the large number of old people with chronic diseases. The number of people living with dementia is expected to rise from around 45 million in 2013 to 136 million by 2050 worldwide with each year bringing around 8 million new cases [2]. The total estimated worldwide cost of dementia was US $604 billion in 2010 and in many cases the costs of informal care account for the majority of these costs. Such costs are around 1% of the gross domestic product of the world’s economy and are set to increase by 85% by 2030 [2].

The use of video surveillance installed in homes of people living with dementia may provide a more economic and efficient means for caring for those occupants who wish to maintain their independent living. For example, such a video surveillance system would be available to family caregivers and would provide a rapid means of ascertaining the wellbeing of their family member with dementia.

However, formal healthcare providers have been reluctant to make use of video surveillance because of ethical concerns in capturing and storing media of people living with dementia as well as others in the home, including formal care staff and other family members that may include other vulnerable people or those who object in principle to video surveillance in their relative’s home [3].

These ethical concerns also more generally relate to damages that can befall our digital world “data-image” with consequent tangible impact on our real world economic, political, social spheres [4]. Stoddart concludes that “it is of the utmost importance that surveillance is truthful; both accurate and appropriate” [4]. These three adjectives would hold value with clear, untampered video surveillance. It would seem that post-processing of video surveillance to mask identity, summarise activity and other operations that offer some form of benefit to the viewer (for example, blurring, pixelating, embossing, silhouetting or using an avatar [5]) may detract from the truthfulness, accuracy and appropriateness of the reality of the person being monitored. It should strive to be ‘congruent with the dignity of persons’ [4].

Another, more complex perspective on the ethics of surveillance posits the ‘just war tradition’ as an ethical framework to be used for decision-making on use of surveillance in a particular instance. In the just war traditional framework for surveillance, justification for such operations can be assessed across a number of principles including reason, authority of the surveillant, declaration of intent, whether surveillance is an act of last resort, the likelihood of success and whether surveillance is a proportionate response [6].

Is there an opportunity for the development of a video surveillance service to provide informal caregivers with a capability to observe the person living with dementia? It is not technically unique to provide such a service to informal family caregivers; a number of existing solutions are available.

One such system worked by recording video when the motion detection system in the camera detected movement in the home of the person living with dementia [7]. This footage was then stored online in a secure location. Depending on how the caregiver had configured their app, they would then receive a real-time notification and the opportunity to view the footage. Users can access these services using smart phones or personal computers at home, work or on the move [7–9] (Lauriks et al. 2010). Another system explored the use of video surveillance to investigate the cause of falls in long-term care [10]. In one strand of this work, the video recorded for the previous 24 h was analysed to seek to identify fall behaviours.

However, these types of services may be difficult for people to use, evoking arguments for and against such a service. The SCIE review on the use of video and associated technology in health and social care settings highlighted the different perspectives of the institution, the ‘resident’ and the caregiver, and how this can alter the relationship and use of the technology for each perspective [11]. The review found that there may be benefits for the person being surveilled in terms of the non-obtrusiveness of the medium, while use of surveillance can impact negatively resulting in ‘self-monitoring’ of behaviour. There are reported benefits for the institution in supporting professionals as they review incidents as well as offering staffing efficiencies. Surveillance also offers both positive and negative impacts on staff behaviour according to research reviewed in the report offering evidence of good and bad behaviour, respectively. While there is some reporting that telecare services including video surveillance offer significant savings [12], the Whole System Demonstrator trials in the UK reported that “telehealth does not seem to be a cost effective addition to standard support and treatment.” [13].

The core issues centre around the utility of the surveillance to the institution and its employees, and the utility to the person being cared for and their circle of caregivers. This paper explores these issues. After describing the background of the impact of dementia on society, the paper describes the related work on the ethical issues of surveillance before describing what a video surveillance system technically comprises. The paper then explains the methodology chosen for a pilot study to gather the views of people living with dementia and their caregivers on surveillance. The results from the mixed methods pilot are presented and discussed.

## Background

Dementia commonly leads to significant physical and mental health problems in those caring for the person living with dementia. Increased caregiver stress is a key trigger in the decision to move a person living with dementia to institutionalised care [14]. It has long been recognised that the impact of caring can lead to mental health problems for the caregiver [15]. National guidelines [16] and regional strategy [17] highlight the importance of support for caregivers in management plans for people living with dementia.

People with dementia and their caregivers perceive that as the disease progresses, the need for health and safety monitoring becomes greater [18]. Assistive Technologies (AT) may be used to support some needs of people living with dementia and support improved quality of life [19]. Caregivers have indicated that AT which increase the person living with dementia’s safety and reduce the caregiver’s anxiety of wandering or accident would be the most favoured [20]. Solutions are sought to make care at home more practical and usable for those at home, primarily because the addressable market for the solutions is growing as people live longer, dementia prevalence is rising and health and social care systems are less able to afford institutional care.

The SCIE report on surveillance technologies in health and social care settings found a ‘notable gap’ in terms of service user and caregiver views on video surveillance [11] indicating perhaps that more research has been carried out on institutional perspectives. Discourse analysis on organising visions on telehealth and telecare has reported four conflicting discourses: humanist, modernist, political economy and change management [21]. The humanist perspective was anchored in the family and personal context, and recognised that technologies could create as well as solve problems. It could be argued that the gap in understanding could be addressed by developing solutions from a humanist perspective to empower families from an early stage looking after relatives with dementia, with tools that help them manage the care required and monitor risks while they are out at work and involved in other aspects of their lives.

## Related Work

In their study of people living with dementia living alone at home, Wattmo et al. [22] concluded, “Increased knowledge of how community-based services can better accommodate the care needs of solitary-living individuals with Alzheimer’s disease is essential.”

There is a significant body of research in the use of video cameras to support people living with dementia (e.g., [8]). Much of it relates to providing ‘tele-presence’ and facilitating remote communication [9, 23, 24], or the use of video to detect and provide electronic notifications about dangerous events such as falls or ‘wandering’ automatically (e.g., [10, 25]).

A study on the use of video surveillance in care facilities revealed four perspectives of use: reflective, real time, reflexive and retrospective monitoring [26]. Reflective monitoring offered senior management a way to understand better the behaviour of residents, while real time monitoring provided, in effect, covert surveillance, described by care co-ordinators in the facilities as a ‘truer perspective’. Reflexive monitoring offered review of incidents that would otherwise not be available for analysis, while retrospective monitoring provided the care facility with a bank of video data to support the decision making of the care management team or assess the quality of care of the care workers. This research concluded that video technology cannot be introduced into a social context without changing the nature of the social environment; concluding that any technology is “socially transformative in nature and should be seen as part of the social landscape, rather than as a separate set of devices or tools for a specific task”.

Socially transformative technology such as video surveillance in the home of people living with dementia raises ethical issues that need to be considered at an early stage as an intrinsic part of the development of any technology-based solutions. Such ethical frameworks must take account of the heterogeneous needs of prospective users including people living with dementia unable to provide consent [3].

In 2014, HC-One, a UK care home organisation carried out a survey of its staff, residents and their relatives after learning that a UK survey found that 80% of British adults supported the use of visible cameras in care homes [27]. The results of their survey showed that support from relatives for visible cameras was 87%, while from residents it was 47% and from staff members it was 63%. Privacy for residents was the largest area of concern across all respondents although other significant concerns included: who has access to footage; who watches the footage; where the footage is stored and how secure it is [28].

Ethical frameworks have been developed to support the ‘dignity, rights, safety and well-being of participants’ [29] and support the following ethical principles or perspectives: autonomy, beneficence, non-maleficence and justice [30]. Using this framework, a camera in the home of a person living with dementia should help that person and their caregivers in terms of supporting the autonomy of both, doing good for the person living with dementia by enabling the caregivers to understand the context of wellbeing (or not) of the person living with dementia at all times remotely, not causing harm to the person living with dementia, and finally offering choice primarily to the caregivers by enabling them to understand the context of wellbeing of the person living with dementia remotely.

Of these four areas, perhaps the most significant is that of not causing harm to the person living with dementia, specifically of invading their privacy. Kenner [31] asks “how might the caregiver’s judgment of the ‘data’ lead to interventions that infringe on the elder’s rights? How is power and control leveraged by scrutinizing and evaluating daily activities against constructed norms?”

Niemeijer et al. [32] pointed out that elaboration of the ethical issues is very difficult and that “there appears to be an inherent duality in the views on using surveillance technology which is rooted in the moral conflict between safety and freedom”. However, this approach of a ‘zero-sum’ mentality between safety versus freedom, assumes mutual exclusiveness of the two perspectives and safety ‘winning’ while freedom ‘loses’, or vice-versa, which is often not necessarily the case. Furthermore, the zero-sum viewpoint can help to erode weaker goals, perspectives, arguments or discourses when set out as competing, for example, safety versus freedom, or privacy versus security [33, 34]. In their work on ‘Privacy by Design’, they present a ‘positive-sum’ paradigm, seeking to avoid these false dichotomies, and have applied this approach in the area of in-home health data collection [34]. Privacy by design does have its detractors, who cite issues around the applicability of this approach when engineering systems, as well as the onus on the system designer rather than the owner of the data [35, 36].

A participatory study with people living with dementia, their family and healthcare professionals found that while those people living with dementia disliked remote monitoring and surveillance, their caregivers were pragmatic, prioritising safety [37].

Kenner [31] summarises the issue succinctly: “exercising control over elderly people with dementia seems to be necessary and may be the best way to ensure wellbeing… if monitoring systems advance further understandings of dementia, help caregivers manage their responsibilities, and keep the elderly safe, how concerned should the public be about issues that have always plagued caregiving relations-control, privacy, autonomy, and power asymmetries?”

The research question arising from these asymmetries relates to the ethical considerations of using assistive technologies that include video surveillance in the homes of people living with dementia, and the implications for a person living with dementia whenever video surveillance is used in their home and access to the camera is given to the person’s family.

## What would a Video Surveillance Solution Look like?

**Fig. 1:**
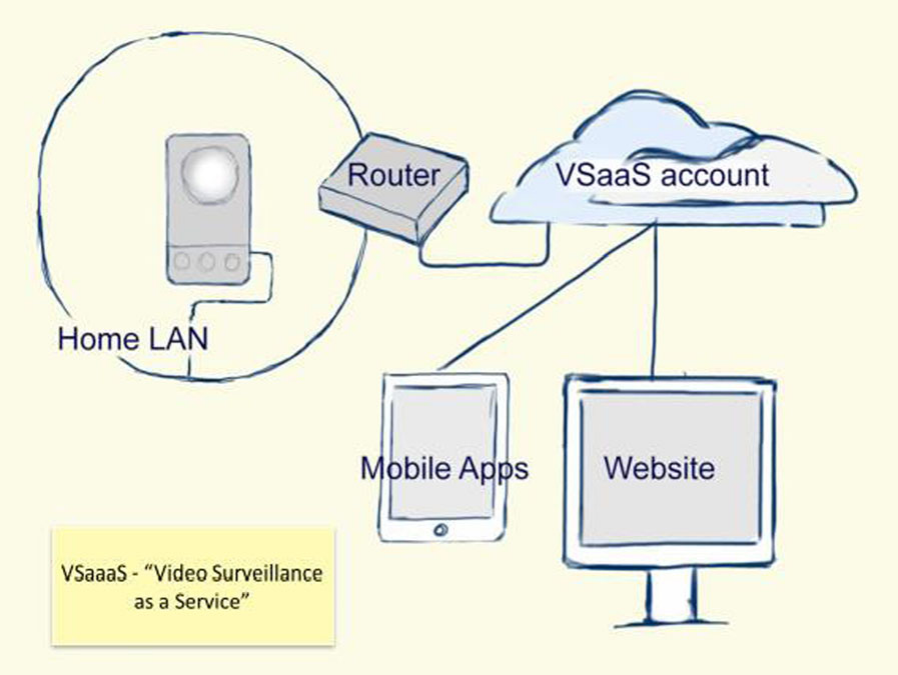
Schematic architecture showing main technical components that comprise the ‘Video Surveillance as a Service’ solution

There is a high market availability of Internet Protocol (IP) video camera with huge variation in price and quality and an emphasis on ease of installation and operation. The co-availability of high quality mobile apps used to view output video offers Video Surveillance as a Service (VSaaS), where the camera connects to a service in the Internet ‘Cloud’. People viewing connect to an account in the cloud rather than connecting directly to the camera, making configuration for use relatively easy and based in the software of the app on the mobile device. From the hardware perspective (Fig. 1), the camera can have a one-click connect button and a unique number that identifies it to the VSaaS solution.

Recorded video does not need to be stored on the camera or anywhere in the home. Instead the recordings are stored in the cloud. If the camera is temporarily unplugged or switched off the VSaaS becomes automatically restored when the camera is switched back on.

**Fig. 2:**
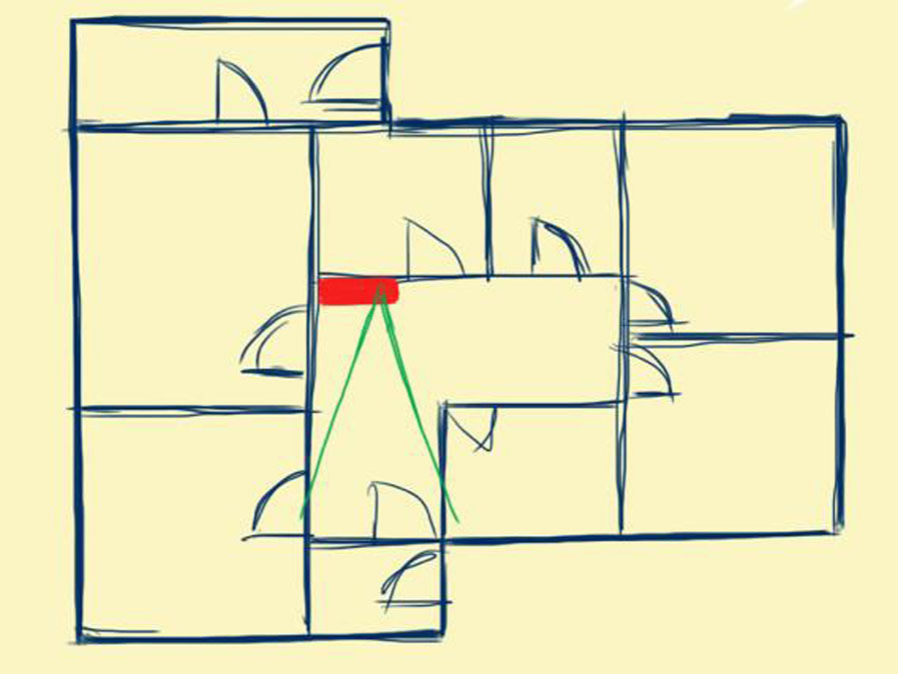
Camera located in non-private central space like a hallway which is good for activity monitoring yet minimises intrusion

**Fig. 3:**
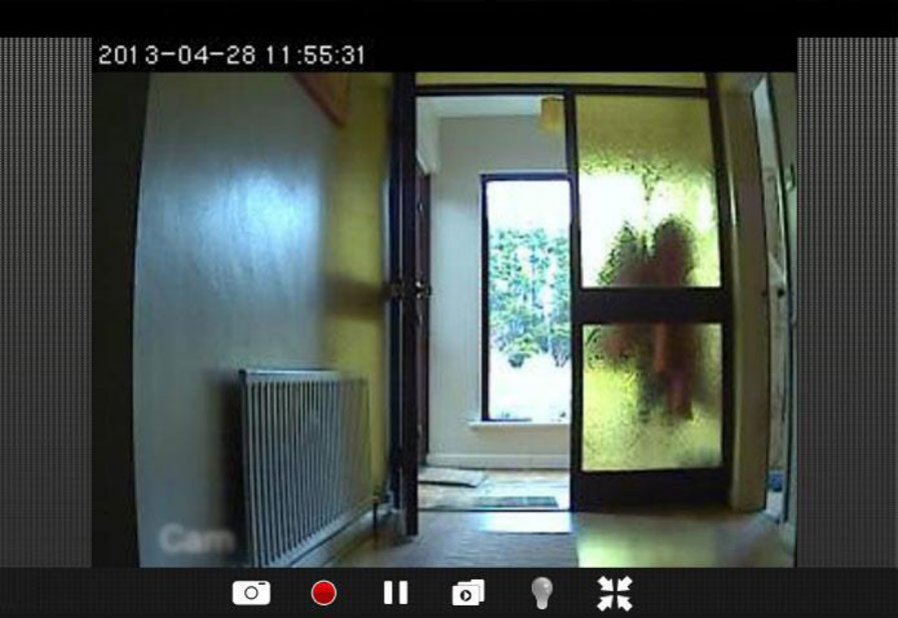
Screen shot of video camera set up as for use in hallway

In terms of location for the camera in a home of a person living with dementia, the hallway (Figs. 2 and 3) of a home, near the broadband router if available and offering a view of the hallway including the front door of the house may be an optimal central location. This offers the ability to surveil activity in a home without intruding on private routines occurring in individual rooms.

**Fig. 4:**
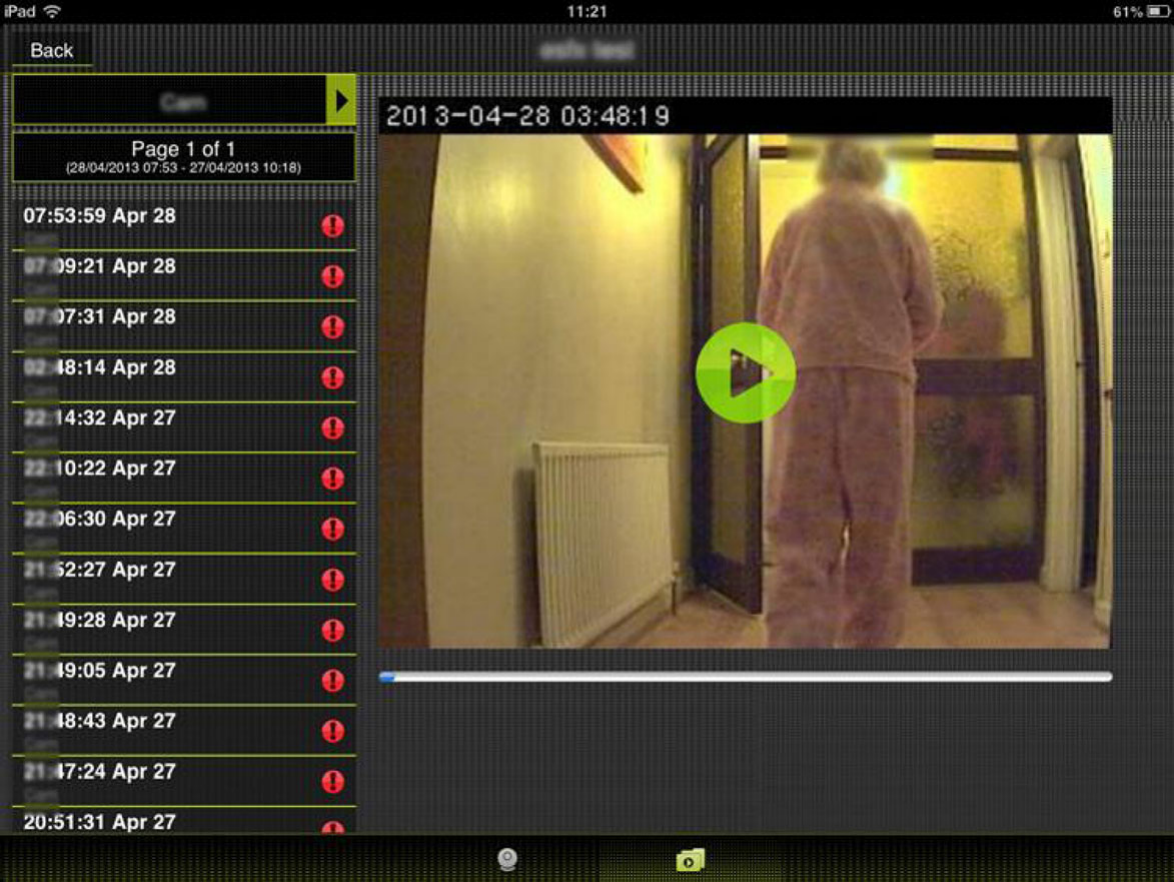
Camera screen shot showing visability of person in hallway

As mentioned, mobile devices are an accessible method in which caregivers can access the video services in real time and via smart notifications. The primary advantage of such mobile access is that the person living with dementia can be conveniently and remotely monitored in real-time by the caregiver via a mobile app (see example in Fig. 4).

The process of ‘checking-in’ on a person living with dementia can be personal and private as it may be carried out using a personally owned smartphone or tablet computer as in the context of keeping the solutions within the family circle.

## Methodology

In order to develop our understanding of the use of video camera monitoring of a person living with dementia at home, we decided to engage with people living with dementia and their caregivers to learn about their views on the technology and its use in the home. The rationale for the study was therefore to elicit the views of both those surveilled and their caregivers, to examine if the literature supported these views and to discuss the findings from the literature with this study group. In total, 24 participants took part in this study, consisting of 2 persons living with dementia, and 22 caregivers. The average age of participants was 56 years (range 22–78 years), 8 (33%) of participants were male and 16 (66%) female.

The *living labs* approach was used to engage with a small group of people living with dementia and their caregivers, using a semi-structured workshop. This approach uses “collaborations of public-private-civic partnerships in which stakeholders co-create new products, services, businesses and technologies in real life environments and virtual networks in multi-contextual spheres” [38]. Living labs offer a “service providing organization in the topic of research, development and innovation” with a set of resources including: areas of competency, local partners and stakeholders, information technology infrastructure, operational methodology and administrative resources [39]. Services in living labs include co-creation [38], which is described as a core service facilitating the development of a product, service or application, decomposed further in to four phases, i.e. addressing the idea, concept, development and the market launch of the product or service [40]. The two areas of ideation and conceptualisation were pertinent to our goals in the workshops, to co-create ideas and concepts relating to video surveillance.

Two evenings workshops were held, facilitated by AgeNI, a charity for older people in Northern Ireland, where those involved participated in discussion. The first event was in AgeNI’s facility in Meadowbank, Omagh, Northern Ireland where 11 people participated and the second was at AgeNI’s building in Belfast where 15 people attended. At each event, a short movie, commissioned to illustrate the concepts proposed was shown and used as a starting point for a general discussion of the issues raised on the benefits or not of using video surveillance [41]. These scenarios formed the basis for the participants’ understanding of how a camera care system might work in practice for a caregiver and their family member living with dementia. The questions that the participants discussed included:

- Can you describe your views on using a video camera in the home?
- What are the three worst things in having a video camera in your home?
- What are the three best things in having a video camera in your home?
- Can you describe a particular situation where a video camera in the home could be important (good or bad)
- Do you think video cameras in the home can be used to support older people with dementia?
- If you could be in charge of designing the use of a video camera in the home, what would your ideas be?

**Fig. 5:**
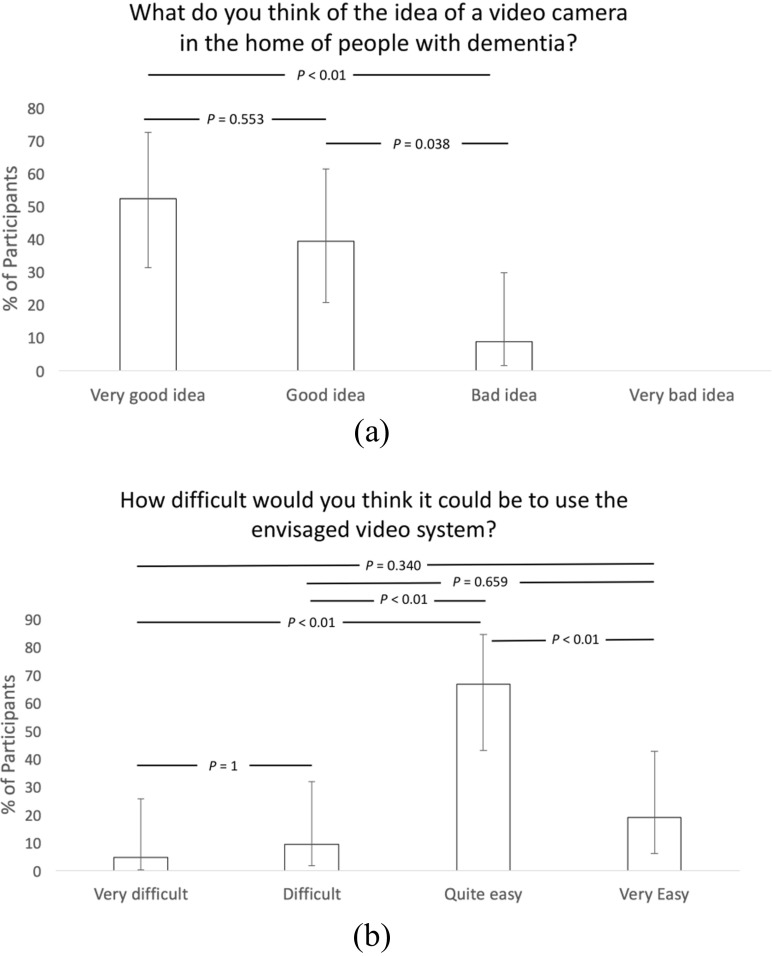
Question responses on (**a**) concept and (**b**) ease of use of camera (Error bars represent 95% confidence intervals and the *p*-values were derived using Chi-square testing in the R programming environment)

**Fig. 6:**
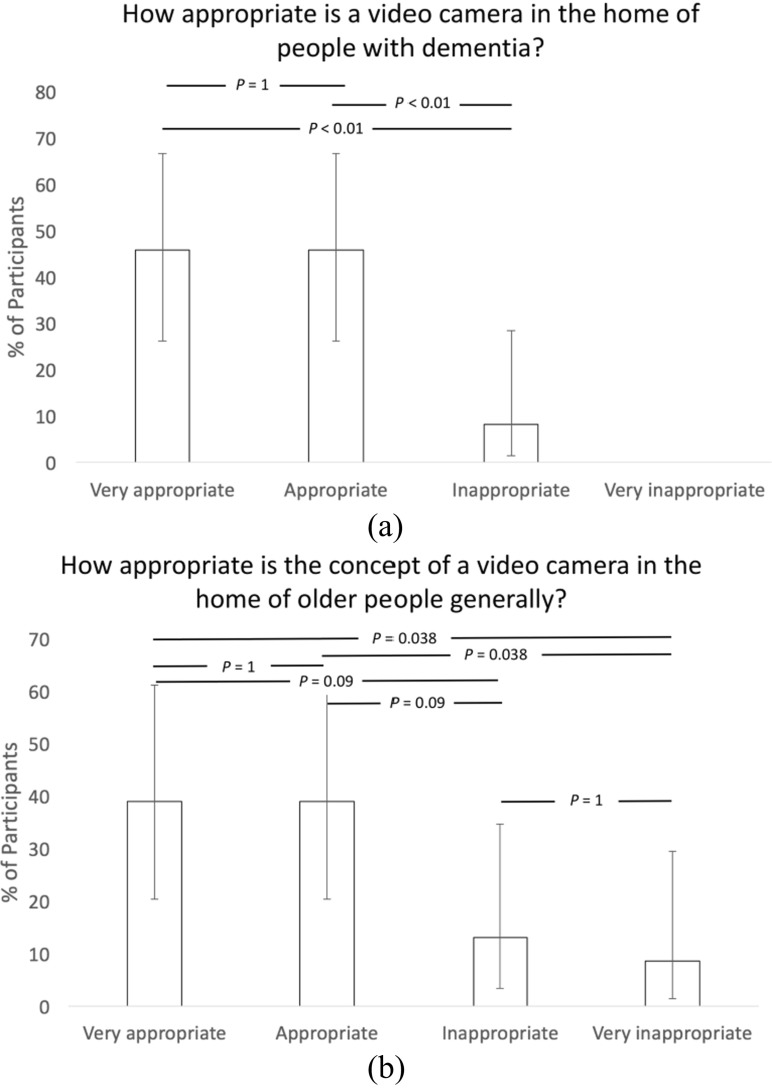
Question responses on (**a**) appropriateness of the concept in home of people living with dementia and (**b**) in home of older people generally (Error bars represent 95% confidence intervals and the *p*-values were derived using Chi-square testing in the R programming environment)

The participants were also asked to respond using a questionnaire which explored issues surrounding the use of cameras as an aid to care for a person living with dementia. The results of these questionnaires are presented in Figs. 5 and 6, and asked the following questions:

- What do you think of the idea of a video camera in the home of people with dementia?
- How difficult would you think it could be to use the envisaged video system?
- How appropriate is a video camera in the home of people with dementia?
- How appropriate is the concept of a video camera in the home of older people generally?

At the workshops, we explored the first two phases of co-creation encompassing the evolution of the idea and the concept of an observation service for caregivers of person living with dementia living at home alone. In doing do, we sought to gather the ideas and perspectives on the concept of a video surveillance system for monitoring persons living with dementia at home.

## Results

The data from the workshops related to questionnaire responses and analysis of the general comments written by the participants. The questionnaire responses reported that 91% thought that the idea of a video camera in the home of a person living with dementia living alone was a very good or good idea (Fig. 5 (a)), while 92% thought that the concept was very appropriate or appropriate (Fig. 6 (a)), dropping to 78% considering it very appropriate or appropriate for use in homes of older people generally (Fig. 6 (b)).

A majority of the participants (86%) reported that actually using the envisaged system would be either very easy or easy (Fig. 5 (b)). In the discussions, there was mention of the system offering to alleviating anxiety of the caregivers. The system would need to be user-friendly and easy to use in order not to introduce new anxieties for the caregivers. The concept was presented as a solution where the person living with dementia would not be required to configure or change it in any way.

The participants supported the concept of camera installation in the house of a person living with dementia with access to the camera given to the person’s family and indicate that the effect on family care for the person living with dementia is complex covering broadly positive aspects as well as more negative aspects, which are discussed in the following paragraphs.

The four prima facie ethical principles are autonomy, beneficence, non-maleficence and justice [30, 42]. The written responses of participants in the workshops reflected these principles in many cases, many presenting arguments and reflections around the net benefit of beneficence with non-maleficence. The principle that presented most frequently related to autonomy, both from the perspectives of people living with dementia:

*“If properly placed being mindful of privacy it allows the person to live at home for longer and remain independent”*

and from the caregivers’ perspective:

*“It relieves the anxiety of the family who are concerned about the safety of the person with dementia and it affords those who are caring for a person with dementia more freedom.”*

Written responses from the participants added more detail. A key phrase analysis of the responses showed broadly positive key phrases reoccurring, such as: ‘feeling of safety’, ‘feeling of security’, ‘reassurance’, ‘peace of mind’, ‘relieve anxiety’, ‘live at home for longer’, ‘remain independent’, and ‘provide support’. More broadly negative phrases that occurred included: ‘consent’, ‘invasion of privacy’, ‘high cost’, and ‘potential for reduced direct input from family’. These responses support the argument of duality between safety and freedom put forward by Niemeijer et al. [32].

The general sentiment from participants can be illustrated in the following quotes.

*“Because it would give me assurance that Mum goes to bed. Also to monitor her movements as she won't wear a telecare bracelet” (participant 11, age 56, female)*

*“If managed appropriately, I feel that this concept could enable someone with dementia to remain at home for longer. Respecting the individual rights is paramount and this concerns me. I think education and demonstration would be beneficial” (participant 6, age 38, female)*

*“If used properly, this could provide great support for those with dementia, provide comfort for those who are caring for them and provide valuable insight into the behaviours that those with dementia exhibit. It could allow relatives living abroad the opportunity of being involved in caring for someone with dementia; like speed cameras, they can change the behaviours of people. Carers who know and are aware of the presence of a camera will afford the person the correct amount of time being paid for and deliver a better quality of care” (participant 7, age 45, female)*

*“Mainly safety, and peace of mind for family” (participant 24, age 78, male)*

There were also more cautious perspectives from participants, for example,

*“Monitoring safety, mobility, bringing peace of mind to carers. Must be suitable circumstances, respecting necessary privacy with proper permissions and for right reasons” (participant 13, age 69, male)*

*“For me it is about the ethics and safeguarding of the vulnerable person. I believe that there are some merits, but I am not totally convinced but can understand that families directly involved would have perhaps divergent views” (participant 5, age 51, male)*

*“If it is limited to movement and hallways I would be more comfortable but in living rooms, bed rooms you are taking away human rights – big issue. Everyone is an individual – even with dementia” (participant 15, age 51, female)*

*“Proper consent from person needs to be sought so that they feel comfortable with this method. System needs to be independent from other government bodies. Only family have access. Should be used by family to eliminate any worries and to identify any support needs to benefit from the care package for the person” (participant 16, age 48, female).*

The final two quotes are perhaps the most interesting. Everyone *is* an individual and has rights. However, obtaining consent from a person living with dementia where the formal care provider therefore also has an ethical obligation of care is difficult and is discussed below.

## Discussion and Conclusions

The review of related work on the introduction of a video surveillance service for people living with dementia living at home has provided significant findings that indicate conditional acceptance of the use of such technology. However, the examination at the institutional level (albeit not the primary focus of this paper) is where the main corpus of the literature on video surveillance of vulnerable people may be found. While telecare does not offer savings at this institutional level [13], the SCIE report indicated that there are both benefits and problems for institutions as well as for those being surveilled [11]. Woolrych et al. explored the use of video surveillance in institutions, finding four perspectives, namely: reflective, real time, reflexive and retrospective, concluding that any technology is “socially transformative in nature and should be seen as part of the social landscape, rather than as a separate set of devices or tools for a specific task” [26].

More generally, the reported ‘notable gap’ in terms of service user and caregiver views on video surveillance indicated a lack of research on video surveillance in the home from the perspectives of the person living with dementia [11]. The four conflicting discourses reported by Greenhalgh include: humanist, modernist, political economy and change management [21] and the humanist perspective has arguably been least adopted at institutional level. It perhaps offers an approach grounded in the context of care for the person living with dementia and their caregiver, and given the different viewpoints from the survey (again on institutional care) between ‘residents’ and caregivers, a humanist approach embedded in the ‘social landscape’ may help to address the gap between these two groups, and support an engagement using video surveillance that is “truthful, both accurate and appropriate” [4].

Our starting point for examining the literature was the ethical framework encompassing: autonomy, beneficence, non-maleficence and justice [30]. In examining these areas, we found views supporting “…an inherent duality in the views on using surveillance technology which is rooted in the moral conflict between safety and freedom” [32]. This indicated a duality in the concept of such a service, between the perspective of protection and safety of the person living with dementia and the utility of such a service in freeing the person living with dementia to live independently at home for longer, offering more convenience and less stress to the informal caregivers. But this ‘zero-sum’ approach has been criticised [33]. Such an approach sets out two competing prima facie goals, where often the principle or goal representing the humanist perspective that is embedded in the social landscape suffers from power asymmetries. Against this approach, the positive-sum paradigm embeds privacy by design to disarm asymmetries and protect the vulnerable when technology is being designed [34]. However, getting privacy by design to work remains challenging. As an example, even if you are paying to have a house built, the builder will not look favourably on daily site visits where you tell them how to ‘do their job’. It is indeed difficult to facilitate the continuing, impactful engagement from people living with dementia and their caregivers in the design and implementation of solutions that work for them in their homes, along with the technical designers and implementers of such solutions.

The workshops with people living with dementia and their caregivers were useful in providing guidance on navigating the ethical issues but were also interesting in that the participants were enthusiastic about discussing the issues, the technology and how it could work. This type of workshops could perhaps help to improve the design and implementation processes for solutions such as video surveillance by promoting the voices of the people living with dementia and their caregivers.

The workshops did not ask specific questions around consent but the most interesting comment on this concept suggested to exclude organisational participation, to keep it within the family and to obtain consent from the person living with dementia being surveilled. There are various consent mechanisms that can be used beyond the person living with dementia giving consent when they have the mental capacity to do so. The concept of advance directives or ‘living wills’, sometimes called a Ulysses contract, is a binding directive provided by the person when they have the mental capacity to provide such a directive [43]. The directive is normally on aspects of future care, and in effect, transfers decision making to a surrogate, for example, a caregiver.

In the UK, the law sets out a single test for assessing if a person lacks capacity to make a particular decision at a particular time [44]. The test is ‘decision-specific’ and a person living with dementia cannot be labelled as ‘incapable’ as a result of their diagnosis. Any intervention must be in the ‘best interests’ of the person living with dementia and again family members can be part of the process of determining ‘best interests’.

The work carried out in this paper indicates that perhaps a clear description of the meaning of video surveillance in practice would be useful to the person living with dementia and their caregivers. Such a description could be articulated in terms of the impact on the lives of the people involved and not just on the technology and its impact at home. The decisions made by people living with dementia could then form part of their living wills, setting out clear boundaries on use of video surveillance on their home.

This idea could be advanced by transforming the privacy by design approach to seek to imbue the views and attitudes of the people living with dementia and their caregivers into the design and configuration of video surveillance services in their homes. This proposed *ethical by design* approach would deliver a toolkit that could be used online, in workshops or one-to-one meetings to deliver informed, personalised consent regarding the use of assistive technology in the home. Perhaps technology could support the gaining and maintenance of consent where capacity is or is likely to be an issue, extending approaches where paper forms are specially adapted to suit the needs of people, as used for people, for example, with Huntington’s disease [45].

Interestingly, the law says that anything done for or on behalf of people without capacity must be the *least restrictive* alternative in terms of their rights and basic freedoms. Does the use of AT, such as remote video surveillance, offer a *less* restrictive alternative to a person’s rights and freedom than moving them out of their home?

The families who participated in this small study have outlined the different perceived effects of video surveillance, including financial, protection, privacy, security, safety, and peace of mind. Those who took part in the workshops generally were supportive of the concept of the use of camera in the homes of people living with dementia, with some significant caveats. A logical next stage is to run a mixed-methods study to gather more evidence on video surveillance of people living with dementia at home.

In conclusion, the use of cameras in the home of a person living with dementia where family caregivers could monitor their family member with dementia was supported as useful, ethical and moral providing the right protocol is in place to gain consent. However, when professional caregivers are involved – either as part of the care team and therefore one of the ‘observed’ by the camera or as part of the authorised ‘observation team’ then the degree of ethical discomfort increases as it would be impossible to gain consent from all possible visitors to a home – however experimenters would often use unavoidable signs to indicate when and where a location is under video surveillance.
